# Effect of sex, age and genetics on crossover interference in cattle

**DOI:** 10.1038/srep37698

**Published:** 2016-11-28

**Authors:** Zhiying Wang, Botong Shen, Jicai Jiang, Jinquan Li, Li Ma

**Affiliations:** 1 College of Animal Science, Inner Mongolia Agricultural University, Hohhot, Inner Mongolia 010018, China; 2 Department of Animal and Avian Sciences, University of Maryland, College Park, MD, 20742, USA

## Abstract

Crossovers generated by homologous recombination ensure proper chromosome segregation during meiosis. Crossover interference results in chiasmata being more evenly distributed along chromosomes, but the mechanism underlying crossover interference remains elusive. Based on large pedigrees of Holstein and Jersey cattle with genotype data, we extracted three-generation families, including 147,327 male and 71,687 female meioses in Holstein, and 108,163 male and 37,008 female meioses in Jersey, respectively. We identified crossovers in these meioses and fitted the Housworth-Stahl “interference-escape” model to study crossover interference patterns in the cattle genome. Our result reveals that the degree of crossover interference is stronger in females than in males. We found evidence for inter-chromosomal variation in the level of crossover interference, with smaller chromosomes exhibiting stronger interference. In addition, crossover interference levels decreased with maternal age. Finally, sex-specific GWAS analyses identified one locus near the *NEK9* gene on chromosome 10 to have a significant effect on crossover interference levels. This locus has been previously associated with recombination rate in cattle. Collectively, this large-scale analysis provided a comprehensive description of crossover interference across chromosome, sex and age groups, identified associated candidate genes, and produced useful insights into the mechanism of crossover interference.

Genetic recombination, a fundamental meiotic process, results in the production of offspring with traits that differ from those in their parents. In eukaryotes, genetic recombination during meiosis leads to a novel set of genetic materials that can be passed on from parents to offspring. Failures in recombination may lead to fatal defects such as aneuploidy^1^,^2^,^3^. In many organisms, crossovers generated by homologous recombination are normally required to ensure proper chromosome segregation during meiosis^4^,^5^. The frequency of meiotic crossovers is a major factor in increasing genetic variability in natural populations and in breeding programs^6^. Previous studies in mammal systems have suggested that crossover frequency can vary both within and between chromosomes, sexes, populations and species^7^,^8^,^9^,^10^. Crossover events are not randomly spaced along a chromosome; instead, they are subject to interference: the presence of one crossover discourages the probability of another crossover event occurring nearby^11^,^12^,^13^. Crossover interference appears to occur as universally as meiosis, which results in chiasmata being more evenly distributed along chromosomes^14^. The number and distribution of crossovers are thus strictly regulated with crossovers/chiasmata formed in optimal positions along the length of individual chromosomes, facilitating regular chromosome segregation^15^.

The level of crossover interference is influenced by many factors, including sex, chromosome length, and possibly age. Studies in humans and mice indicated that crossover interference differs between the two sexes, with a stronger interference in female than in male mice^16^. However, the degree of interference in humans was recently inferred to be lower in females than in males^5^. Broman and Weber reported that the interference parameters of some chromosomes in females were higher than that in males^17^. Another study used two-pathway model to estimate crossover interference in humans, which demonstrated that the interference in females was stronger than that in males^18^. Several studies indicated that the degree of interference on a chromosome can be reasonably well predicted by the chromosome map length^4^,^19^,^20^,^21^. Apart from that, some studies suggested that maternal age had a significant effect on the frequency of crossover^22^,^23^. However, the link between crossover interference and paternal age remains elusive^24^,^25^.

Generally, the intensity of crossover interference decreases with distance between crossovers. Crossovers on the same chromosomal arm interfere more strongly than those on two arms even with the same distances in between^26^. One report suggested that interference depends more on genetic distances than physical distances^27^. And most of studies obtained the interference parameters based on genetic distances between double crossovers^17^,^18^,^28^. Interference acts across widely varying distances in different species: tens of kilobases (Kb) in budding yeast and tens of megabases (Mb) in mice^27^,^29^. In female and male mice, crossover interference had peak values at 40 Mb and 57 Mb double-crossover distances, respectively^16^. A study in Drosophila found no evidence of interference when the distance between two crossover intervals is greater than 46 cM^30^.

Coefficient of coincidence, expressed as a ratio of observed to expected double recombinants subtracted from 1, is a traditional measure of interference^31^,^32^. In the past decades, a gamma model was often applied to estimate interference parameters, which assumes that all crossover events are subject to a same interference process^33^,^34^,^35^. Nonetheless, evidence from some human studies suggested the existence of a subset of crossover events escaping interference^5^,^8^. As such, the Housworth-Stahl “interference-escape” model was adopted, which assumes the distances between crossovers as a mixture of two processes^13^. In one process, crossovers are distributed as a gamma model, whereas in the other process, crossovers are distributed randomly without interference. Crossover interference can be classified into two types, positive and negative. With positive crossover interference, the occurrence of a crossover event reduces the likelihood of a second crossover event occurring in the same vicinity; however, under negative crossover interference, a crossover in a particular region enhances the occurrence of other crossovers in the same region of a chromosome^36^,^37^,^38^. Typically, positive crossover interference is more common in meiotic organisms^33^,^37^,^39^, although exceptions do exist^40^,^41^.

In this study, we fitted a Housworth-Stahl “interference-escape” model to millions of crossover events to estimate crossover interference parameters in female and male meioses for two cattle breeds, Holstein and Jersey. This large-scale analysis allows us to investigate crossover interference as a function of breed, sex, chromosome length and parental age. Previous studies have found that, like recombination rate, interference is also partially under genetic control^9^,^42^,^43^,^44^. Using interference parameters as phenotype, we also performed a genome-wide association study to find genomic regions and candidate genes related to crossover interference in cattle.

## Results

### Distribution of crossover events across chromosomes in Holstein and Jersey cattle

By constructing three-generation families from large cattle pedigrees, we inferred crossover events for paternal meiosis from a sire/offspring pair and for maternal meiosis from a dam/offspring pair. In Holstein, we detected 1,662,714 crossover events in 71,687 dam/offspring pairs and 3,668,801 crossover events in 147,327 sire/offspring pairs. As shown in Supplemental Tables 1 and 2, a linkage map of all 29 *Bos taurus* (BTA) autosomes had a total genetic length of 2,519 centi-morgan (cM) in males and 2,372 cM in females. Consistent with previous reports^44^, male-biased genetic map lengths were observed across all the 29 autosomes (Male: Female ratio in map length = 1.06; Supplemental Fig. 1). For Jersey cattle, we identified a total of 732,611 crossover events in 37,008 dam/offspring pairs and 2,334,354 crossover events in 108,163 sire/offspring pairs (Supplemental Tables 3 and 4). The sex-specific map lengths across all 29 autosomes were 2,369 cM and 2,229 cM in males and females, respectively, indicating a consistent male-biased heterochiasmy across cattle breeds (Male: Female map length ratio = 1.06; Supplemental Fig. 1).

The total number of crossovers showed a decline with chromosome size, due to the decreasing physical map lengths across the chromosomes (Supplemental Fig. 2). Although the relative frequencies of zero, single, double and triple crossovers were markedly different (Supplemental Tables 1–4), the trends of the four types of crossover events across 29 chromosomes are consistent for the two sexes (Supplemental Fig. 3). As expected, the proportion of meiosis with no crossovers on a chromosome is higher for smaller chromosomes. There is a large proportion of meiosis with single crossover, accounting for more than 40% for all chromosomes. The relative frequencies of double and triple crossover meiosis decrease with chromosome size, contributing to the decline in total number of crossovers. When comparing the two sexes, females have a slightly lower frequency of double crossovers and more zero-crossover events than males, which likely explains the smaller total number of crossovers in females. However, the frequencies of single and triple crossovers are similar in the two sexes. Note that the proportion of triple crossovers is very low for both sexes. Except for zero crossovers, the relative frequencies of single, double and triple crossovers in Holstein are higher than those in Jersey. The Holstein cows and bulls had 1.60 and 1.45 times higher frequencies of double or triple crossovers across all the autosomes than that of Jersey, respectively (Supplemental Table 5).

### Estimation of crossover interference parameters in two cattle breeds and two sexes

To characterize crossover interference in cattle, we applied the Housworth–Stahl interference escape model to a large number of crossover intervals and estimated two crossover interference parameters, interference (*v*) and escape (*p*)^5^,^13^,^35^. By modeling the distribution of inter-crossover distances across the autosomes in each subgroup separately, we estimated crossover interference parameters in the two cattle breeds and in the two sexes, respectively. Note that crossover escape refers to the number of crossovers that appear to escape interference. For Holstein, both of the interference and escape parameters are larger in females than in males (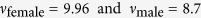;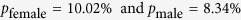). Consistent results are observed in Jersey (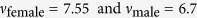; 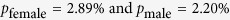). Interestingly, the estimates of interference and escape parameters in Holstein are both higher than those in Jersey.

**Figure 1:**
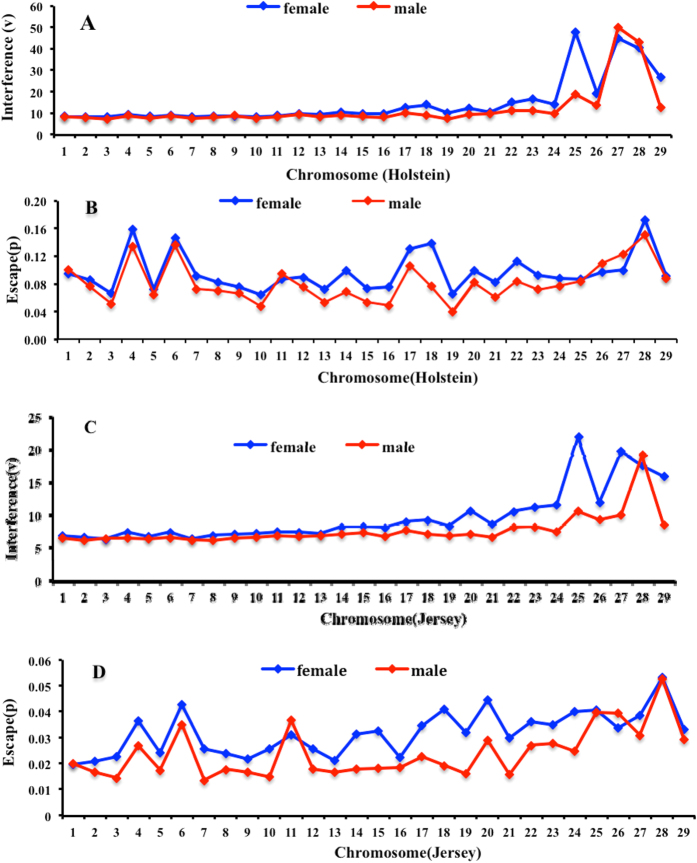
Crossover interference parameters across 29 bovine autosomes. (**A**) Per-chromosome estimates of the interference parameter in Holstein as obtained from the Housworth–Stahl interference escape model. (**B**) Per-chromosome estimates of the proportion of events escaping interference in Holstein as obtained from the Housworth–Stahl interference escape model. (**C**) Per- chromosome estimates of the interference parameter in Jersey as obtained from the Housworth-Stahl interference escape model. (**D)** Per-chromosome estimates of the proportion of events escaping interference in Jersey as obtained from the Housworth-Stahl interference escape model.

**Figure 2:**
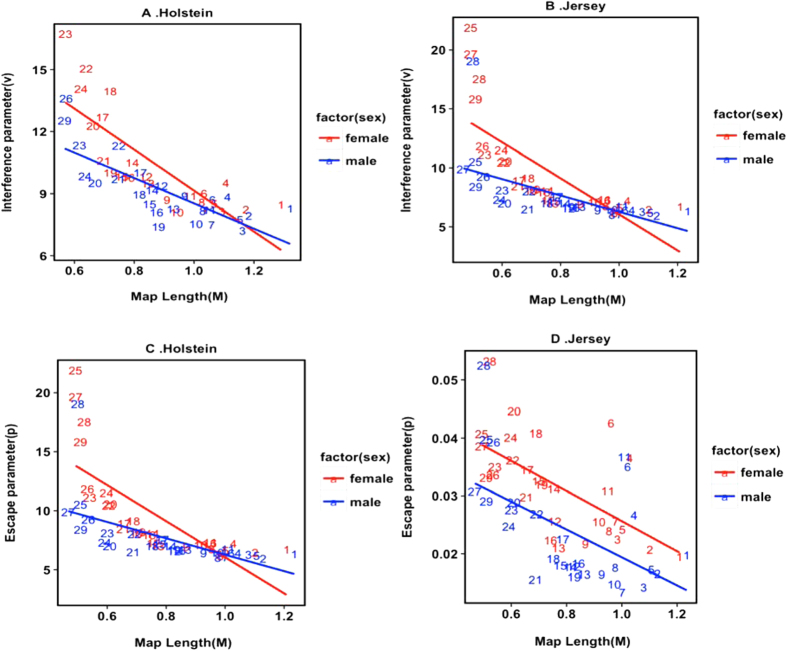
The relationship between chromosome map length and the interference parameter (ν) in Holstein (**A**) and Jersey (**B**), and between map length and the escape parameter (p) in Holstein (**C**) and Jersey (**D**). Linear fits are shown for females^7^ and males (blue).

To evaluate how crossover interference changes with chromosomes size, we estimated crossover interference and escape parameters for each chromosome. The evidence indicated that the degree of interference and escaping interference varies across chromosomes (Fig. 1 and Supplemental Tables 6–7). We found that estimates of the interference parameter were higher on shorter chromosomes. For Holstein, the strength of interference can be reasonably well predicted by the chromosome map length (Fig. 2 and Supplemental Table 1). This relationship is significantly different between males and females (*P* < 0.001). Some small chromosomes appear to have very high degrees of interference, with notable outliers being chromosomes 25, 27–29 in females and 27–28 in males, respectively (Fig. 2). Instead, the fraction of events that escape interference exhibits a non-significant relationship with chromosome map length (Fig. 2 and Table 1). Largest estimates of escape parameter are observed on chromosomes 4, 6, 17, 18 and 28 in females and on chromosomes 4, 6, 17 and 28 in males, respectively. For Jersey, there are significant linear relationships between both interference and escape parameters and chromosome map length for the two sexes (Fig. 2 and Table 1). The squared correlation coefficients between interference and map length are 0.57 in females and 0.40 in males, respectively. Note that the estimated interference parameters are extremely large for chromosomes 25, 27–29 in females and for chromosome 28 in males (Fig. 2). Conversely, we found that the escape parameter has a strong, negative relationship with chromosome map length (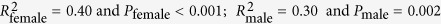). Overall, the results showed that the strength of both interference and escape parameters are stronger in females than in males, especially for smaller chromosomes, which may be related to the smaller total number of crossovers in cows than in bulls.

### Genetic control of crossover interference in males and females

**Figure 3:**
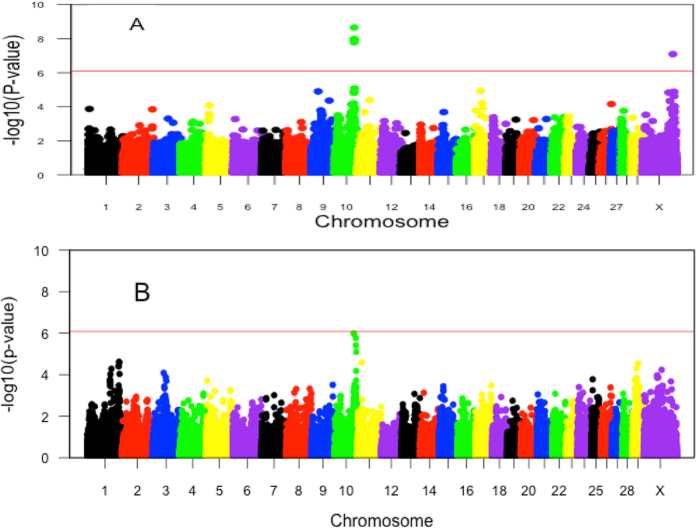
Manhattan plot of the GWAS of the interference parameter for males (**A**) and for females (**B**) in Holstein. Different colors were used to distinguish the 30 chromosomes. The genome-wide significance level of 8.3 × 10^−7^ was shown by the red horizontal solid line.

Based on the distribution of inter-crossover distances in their offspring, we estimated crossover interference parameters for sires with more than 15 offspring and for cows with over three offspring. Using interference and escape parameters as phenotype, a total of 1,125 bulls and 4,032 cows were included in the GWAS analyses for Holstein. The estimates of interference parameter range from 6 to 12 in bulls and from 5 to 15 in cows, respectively (Supplemental Fig. 4). The escape parameter values for most of animals were less than 0.1. Using a linear mixed model, we tested the association between genome-wide interference and escape levels and 60,577 SNPs in males and 60,582 SNPs in females. We adopted standard errors that were obtained from the estimation of interference parameters as residual variances in the mixed model, and used a genome-wide significance level of 8.3 × 10^−7^ by the Bonferroni correction. A total of five SNPs in a locus on chromosome 10 were identified to be significantly associated with interference parameter in bulls (Fig. 3 and Table 2). On the contrary, no SNPs were detected to be associated with escape interference parameter in males. We found no SNPs associated with female interference or escape interference parameters at the genome-wide significance level, but noticed an association with interference parameter on chromosome 10 that almost reaches genome-wide significance (Fig. 3). Interestingly, the locus associated with interference on chromosome 10 is shared between bulls and cows (Fig. 3). The top SNP at this locus, BTA-78285-no-rs, has been previously associated with recombination rate in both males and females^44^. The nearest gene is *NEK9*, which is related to spindle organization, chromosome alignment and cell cycle progression^45^. To better understand the association between this QTL and two recombination traits, recombination rate and crossover interference, we conducted additional association tests between crossover interference and this locus conditional on recombination rate. This conditional analysis revealed decreased levels of significance for the association of this QTL with crossover interference, but the top association still appeared at SNP BTA-78285-no-rs (*P*_Conditional_ = 1.3 × 10^−5^).

The same approach was implemented to perform GWAS of both interference and escape parameters for 734 bulls and 860 cows in Jersey. Compared with Holstein results, the levels of interference in both sexes were lower, with the majority of interference estimates distributed between 5 and 10 in bulls and between 5 and 8 in cows, respectively (Supplemental Fig. 4). The estimates of escape interference in Jersey were also smaller than those in Holstein, most of which were less than 0.05 in both sexes. Although we found no SNPs associated with interference or escape in Jersey at the genome-wide significance level, there is a nominally significant association at the locus on chromosome 10 that was associated with interference in Holstein bulls (Supplemental Fig. 5). To boost the power of the association test, we combined Holstein and Jersey samples together to a meta-analysis of the interference parameter. A total of six SNPs on the chromosome 10 locus were identified to have a significant effect on crossover interference in males, with the strongest association at BTA-78285-no-rs (*P*_meta_ = 9.2 × 10^−12^; Supplemental Fig. 6 and Supplemental Table 8).

### Genetic control of inter-crossover distances in males and females

As interference levels were primarily determined by inter-crossover distance, we can directly use this metric for GWAS. Likewise, bulls that had more than 15 progeny and cows with over 3 progeny were included. Due to the impact of varying chromosome lengths, inter-crossover distances were standardized by chromosome length (RCO). Then, the RCO of an individual was obtained by averaging RCOs across all the autosomes. Although no SNPs were associated with RCO in each breed individually, we identified one SNP on chromosome 10 to be associated with RCO in males when Holstein and Jersey samples were combined together into a meta-GWAS (Supplemental Fig. 7). Importantly, the top SNP associated with inter-crossover distance is SNP BTA-78285-no-rs, confirming the association between this SNP and crossover interference.

### Trends in crossover interference parameters over maternal age groups

**Figure 4:**
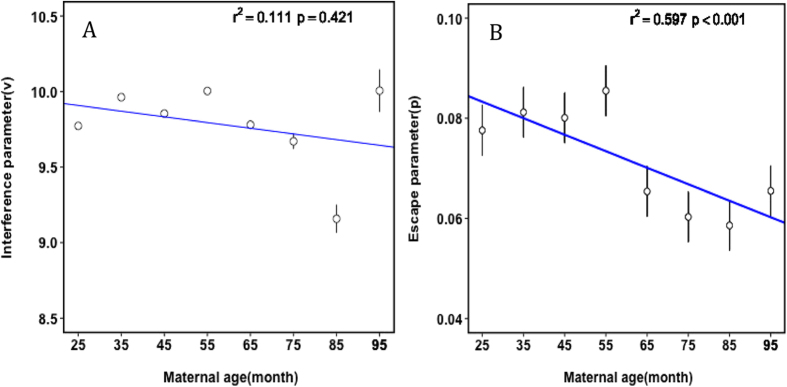
Crossover interference (**A**) and escape (**B**) parameters as a function of maternal age in Holstein. Error bars show 95% confidence intervals. Due to the large sample size, the errors of interference are close to zero for groups 65 month old or younger.

Previous research has indicated a relationship between maternal age and the number of recombination events^5^,^46^,^47^, although the direction of this correlation is inconsistent. Interestingly, many studies reported no effect of paternal age on recombination^5^,^24^,^46^. To investigate whether crossover interference changes with maternal age in cattle, we divided the Holstein cows into eight groups based on age (Supplemental Table 9), and fitted a Housworth–Stahl interference escape model to each group separately. We observed a striking decline in the proportion of events that escape interference with maternal age (Fig. 4A; *R*^2^ = 0.597 and *P* = 9.56 × 10^−6^), decreasing from 8.55% for cows under 5 years old to 5.86% for cows over 7 years old (Supplemental Table 9). However, there is no such correlation between maternal age and the degree of interference (Fig. 4B).

One potential concern is that the degree of interference may be affected by recombination rate. As the distribution of distances between crossovers depends on the number of crossovers, the distance between two crossovers is shorter when more crossovers occur in one chromosome. Hence, if there is a change in the recombination rate with age, it may influence the interference estimates^5^. To account for the potential confounding effect of recombination rate, we conducted a stratified sampling analysis. We extracted data from chromosome 1 in all female meiosis data. Specifically, for each age group, we subsampled individuals in order to ensure that each subgroup has exact the same distribution of number of crossovers per meiosis. Finally, a total of 679 individuals in each subgroup were analyzed. This subsampling was repeated ten times for each age group. We found that there is an obviously decrease trend in the escape parameter (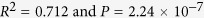), but only a marginal significant effect of maternal age on crossover interference was discovered (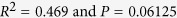).

## Discussion

With the development of high throughput genotyping technologies and modern statistical methods, genomic selection had been widely applied to improve dairy and beef production in cattle, resulting in dramatic genotypic and phenotypic changes compared to the traditional breeding^48^,^49^. Understanding the mechanism of genomic selection for cattle, including recombination and cattle genetic maps, will be helpful for the improvement of cattle genetics^50^,^51^. In this study, we make full use of the genotype information from large pedigrees to obtain genetic maps for both males and females in Holstein and Jersey cattle, locate crossover positions, fit Housworth-Stahl ‘interference escape’ models to estimate the degree ‘of crossover interference on each chromosome and the whole genome, and then investigate if maternal age had an impact on crossover interference.

Based on the genetic maps of the two sexes, we found male-biased heterochiasmy in both Holstein and Jersey cattle. The ratio of male to female map length in our study (1.06:1) is slightly higher than the ratio of 1.02:1 reported by Steven M. Kappes *et al*.^52^. For all, the current maps are longer than the previous study from Ma *et al* 53, which is possibly related to an increased marker density. For the relative frequencies of zero, single, double and triple crossovers on a chromosome, we found no significant difference between females and males regardless of Holstein or Jersey cattle, while these proportions were markedly different from a mouse study by Petkov *et al*.^16^. However, the small difference in sex-specific maps and the distribution of crossovers does not preclude sex-specific differences in certain regions of the genome^52^, so all our analyses were performed on a sex-specific manner. In addition, the ratio of meiosis with no crossovers on a chromosome was higher for smaller chromosomes, and the frequencies of double and triple crossovers decreased with the length of chromosomes. The trend in relative frequencies of zero to triple crossover events across chromosomes was consistent with studies in mice^16^,^34^. A simple test for interference suggested that if there is interference, then the ratio of non-crossover classes to singles should differ significantly from the ratio of singles to doubles^54^. Obviously, our data showed that crossover interference existed in both sexes for two cattle breeds.

We fitted the Housworth–Stahl interference escape model to estimate crossover interference levels in Holstein and Jersey cattle. Both interference and escape parameters were higher in females than in males, especially for small chromosomes. Interestingly, this sex difference in the interference parameter in cattle is opposite to human results, but the difference in escape parameter is consistent between cattle and humans^5^,^35^. Another two reports showed that the levels of interference and escaping interference on some chromosomes are stronger in females than in males^13^,^39^. Some studies supposed that the interference level was influenced by the recombination rate^55^; nevertheless, individual level variations in interference indicated that the individual differences in recombination do not fully explain the observed differences in interference^39^. There is a clear trend of shorter chromosomes having higher interference parameters regardless of sex; by contrast, no such relationship is seen in the fraction of events that escape interference, which is consistent with some reports in humans and mice^4^,^5^,^34^. Kaback *et al*. showed, in yeast, that the size of a chromosome may have a causal effect on the level of recombination, and this may be the result of size-dependent control of the level of interference^56^. Of note in both sexes, the estimate of interference parameter on some chromosomes appears to be extremely large, such as chromosomes 25 to 29. Similar findings in humans have been reported previously, reflecting the fact that low frequencies of double crossovers and shorter genetic distances between double crossovers on these chromosomes were among the main reasons that produced these outlier values^5^,^34^.

GWAS has been performed for recombination rate in mammalian species, and SNPs were reported to have significant effects on recombination rate. Ma *et al*. identified SNPs on chromosomes 1, 3, 6, 10 and 26 that had significant influence on cattle recombination rate, and most of the associations were shared in both sexes^44^. And some genes, such as *RNF212* and *PRDM9*, were associated with recombination rate across species^57^,^58^,^59^. Crossover interference, another feature of recombination, has been rarely considered in GWAS up to now. A study by Sandor *et al*. found no QTL to have an effect on interference levels in cattle when the crossover distances were measured in centi-morgans^43^. In our study, we found a few SNPs near the *NEK9* gene on chromosome 10 had significant effects on interference and double-crossover distances in bulls. Interestingly, this genomic region has been associated with recombination rate in both sexes in cattle^44^.

Previous reports have suggested that maternal age had a significant effect on recombination rates, but no such relationship was observed between paternal age and recombination features^5^,^47^. Most studies in humans showed a jump in the number of crossovers for a maternal age of 30 to 35^5^,^22^. It was assumed that the apparent increase in the number of crossovers with maternal age was due to a selection effect, such that eggs with more crossovers were more likely to give rise to offspring. Indeed, the number of crossovers had a directly association with crossover interference levels^47^. On the contrary, the frequency of crossovers was reported to decrease with maternal age in other species^60^. To better understand the effect of maternal age on recombination, we studied the interference levels in female meiosis across age groups. Our results indicated that there were decreasing trends of both interference and escape interference in the two breeds of cattle, whereas the association between escape parameter and maternal age was statistically significant in Holstein. In addition, we showed that there was an increase in the interference level for cows less than 5 years old, and then a decreasing trend was observed after 5 years of age. Campbell *et al*. explained that if the number of crossovers increases, the distances between them are necessarily shorter, which may in turn influence the interference parameter estimates^5^. Although several explanations have been previously proposed, including genotyping errors and parental age effect on recombination rate, the mechanism for the decreasing or increasing trend of crossover interference with maternal age remains unclear.

## Methods

### Crossover identification in cattle pedigree

Based on our previous study, we phased the genotypes in three-generation families that are extracted from the full cattle pedigree^44^. Within a three-generation family, the genotype of an animal was phased based on the parent genotypes, and crossovers locations were identified by comparing one haplotype of the offspring (paternal or maternal) to the two haplotypes of the corresponding parent (sire or dam). In each family, we inferred recombination events for a paternal meiosis and a maternal meiosis. Note that the recombination events defined here are observed crossovers. To construct recombination maps, we calculated recombination rate between consecutive SNPs based on the observed number of crossover events. We used the recombination rate between adjacent SNPs as genetic distance, since map distance in centi-Morgan is almost equal to recombination rate times 100^19^. Here we only included the highest-quality or most informative meioses where the offspring, the parent, and the grandsire were genotyped by at least 50 K SNP chips. We also removed meioses that have more than three crossover events on one chromosome. After filtering, the maximum number of crossovers on each individual chromosome was restricted to three. In addition, we excluded chromosome X in all our analyses due to the poor quality of the current genome assembly on X.

### Inferring crossover interference

We fitted a Housworth–Stahl (HS) interference-escape model for each autosome and for all autosomes combined. Previously studies indicated that the HS model is a better model to fit the crossover data than others, such as the Gamma model and the Count-location model^5^,^13^,^39^. The HS interference-escape model is a mixture of two components: one component assumes that crossovers occur with an interference parameter (υ) and the other supposes it occurs with no interference (υ = 1). Let 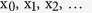 be the genetic distances between adjacent chiasmata, where x_0_ is the distance from the p-terminal end of a chromosome to the first chiasma. The distances between chiasmata are given by a gamma distribution with shape *υ* and rate 2*qυ*. As such, the density of x_*i*_ is 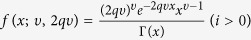; and the density of x_0_ is 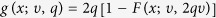, where *F* is the cumulative distribution function (cdf) of 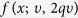. However, the actual chiasmata locations are not directly observed. Rather, only the crossovers derived from chiasmata positions are obtained. Let 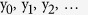 be the genetic distances (Morgans) between adjacent crossovers. Each y_*i*_ is independent to others, with a density function 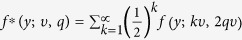. Likewise, the density of y_0_ is given by 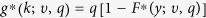, where *F* ^*^ is the cdf of *f* ^*^. Likewise, let G^*^ represents the cdf of g^*^.

Now consider a dataset from a single meiosis where the inter-crossover distances are given by 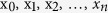, where 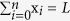. We assume these events are derived from two types of crossovers. To calculate the likelihood of the data, we must sum over the 2^*n*^ possible ways of assigning crossovers to the two types. We split the data into two sets of inter-crossover distances, 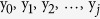 for the interference-free type and 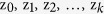 for the interference type, where 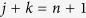. The likelihood of the data from the interference-free type is:


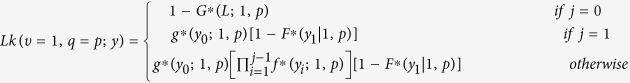


And the likelihood of the data from the interference type is:


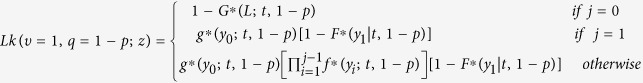


Maximum likelihood estimation of two parameters (*υ* and *p*) was calculated using the Nelder-Mead method in MATLAB (https://github.com/auton1/interference/)^61^. In addition, the R package (xoi) was also used to estimate the crossover interference levels^34^.

### GWAS of crossover interference

The interference and escape parameters of each animal were estimated by applying the HS interference-escape model to the crossover distances in the offspring across all autosomes. Due to the intensive use of artificial insemination in cattle breeding, males have more progeny than females. In this analysis, we included bulls with over 15 offspring and cows with more than 3 progeny. A total of 1,125 bulls and 4,032 cows in Holstein were included in the GWAS analyses of crossover interference. Considering the interference or escape parameter as phenotype, we tested for association between phenotype and 60,577 SNPs in males and 60,582 SNPs in females using a linear mixed model. We used standard errors obtained in the HS model fitting as residual variances and a Bonferroni corrected genome-wide significance level (0.05 divided by the number of SNPs). The same approach was applied to Jersey cattle, including 734 bulls (58,418 SNPs) and 860 cows (58,874 SNPs). To increase the power of GWAS, we also combined the Holstein and Jersey data together to conduct a meta-GWAS of interference and escape parameters. The linear mixed model was fitted as following,


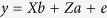


where *y* is the observation of each individual, and X is incidence design matrices relating records of the trait to fixed effects (*b*) that include a population mean and the additive effect of the candidate SNP. *Z* is a design matrix for a random animal effect (*a*) and *e* is residual error. The MMAP software was used for all GWAS analyses^62^. The R package (qqman) was applied to generate Manhattan plots^63^.

As interference levels were primarily determined by inter-crossover distances, we also used this metric for GWAS analysis. Because the autosomes vary by total length, double crossovers distances were measured in cM and standardized as ratios of CO to chromosome map length (RCO). Then the RCO of each progeny was obtained by averaging RCOs across all the autosomes. And the average RCO of all progeny from each parent was used as phenotype. A mixed model was used to test the association between SNPs and RCO in each of the two sexes separately.

### Maternal age effect on interference and escape parameters

To investigate whether crossover interference changes with maternal age, we subdivided the Holstein data into eight quantiles based on maternal age (Supplemental Table 10) and fitted the HS interference-escape model to obtain estimates of interference and escape parameters in each age group separately. One potential concern is that the degree of interference may be influenced by recombination rate. To clarify this concern, we conducted a stratified sampling experiment using chromosome 1 data in females. Specifically, for each age group, we subsampled individuals to ensure that each decile has exactly the same distribution of the number of crossovers per meiosis. First, for each age group (e.g. group *i*), we counted the number of individuals with *x* crossovers, which we call *Ni(x*). For each possible value of *x*, we estimated the minimum *Ni(x*) across all decile age groups, so that 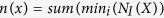. We then subsampled individuals within each decile by randomly selecting *n(x*) individuals, without replacement, for *x*. Finally, a total of 679 and 570 individuals were analyzed across maternal age groups. We repeated 10 times for each age group in the analysis. To study whether sample size has an impact on the estimation of crossover interference parameters, we extracted data from the first maternal age group to perform another sampling analysis with varying sample sizes from 1000 to 10,000. The results indicated no difference for both interference and escape parameters among the samples.

## Additional Information

**How to cite this article**: Wang, Z. *et al*. Effect of sex, age and genetics on crossover interference in cattle. *Sci. Rep.* **6**, 37698; doi: 10.1038/srep37698 (2016).

**Publisher's note:** Springer Nature remains neutral with regard to jurisdictional claims in published maps and institutional affiliations.

## Supplementary Material

Supplementary Information

## Figures and Tables

**Table 1:** Relationship between chromosome length and interference and escape parameters in females and males for Holstein and Jersey cattle.

Breed	Sex	Parameter	Intercept	Slope	*P*-value	*R*^2^
Holstein	Female	Interference	19.05	−9.91	<0.001**	0.6457
		Escape	0.11	−0.02	0.50^ns^	0.0210
	Male	Interference	13.73	−5.45	<0.001**	0.5135
		Escape	0.09	−0.01	0.61^ns^	0.0146
Jersey	Female	Interference	21.35	−15.30	<0.001^**^	0.5706
		Escape	0.05	−0.03	<0.001^**^	0.3960
	Male	Interference	13.22	−6.95	<0.001^**^	0.3680
		Escape	0.04	−0.02	0.002^*^	0.2977

**Table 2:** SNPs associated with interference parameter in males for Holstein.

SNP_rs	SNP	Chr	Position	Freq	Beta	*P*-value
rs43640523	BTA-78285-no-rs	10	86717378	0.53	0.66	2.2 × 10^−9^
rs41591947	UA-IFASA-7857	10	86774733	0.45	−0.64	1.0 × 10^−8^
rs43643710	BTB-00438757	10	86956123	0.36	0.66	1.2 × 10^−8^
rs41257023	Hapmap57084-ss46526565	10	87016969	0.64	−0.66	1.2 × 10^−8^
rs43640475	BTB-00438922	10	86679535	0.35	0.65	1.7 × 10^−8^
