# Supplementary material for: Effect of sex, age and genetics on crossover interference in cattle
Source: Sci Rep. 2016 Nov 28;6:37698. doi: 10.1038/srep37698 (PMC5125268; doi:10.1038/srep37698)
Supplement: Supplementary Information [file srep37698-s1.pdf]

Effect of sex, age and genetics on crossover interference in cattle

Zhiying Wang[1,2], Botong Shen[2], Jicai Jiang[2], Jinquan Li[1,*], Li Ma[2,*]

[1] College of Animal Science, Inner Mongolia Agricultural University, Hohhot, Inner Mongolia 010018, China

[2] Department of Animal and Avian Sciences, University of Maryland, College Park, MD, 20742, USA

Supplemental Figures

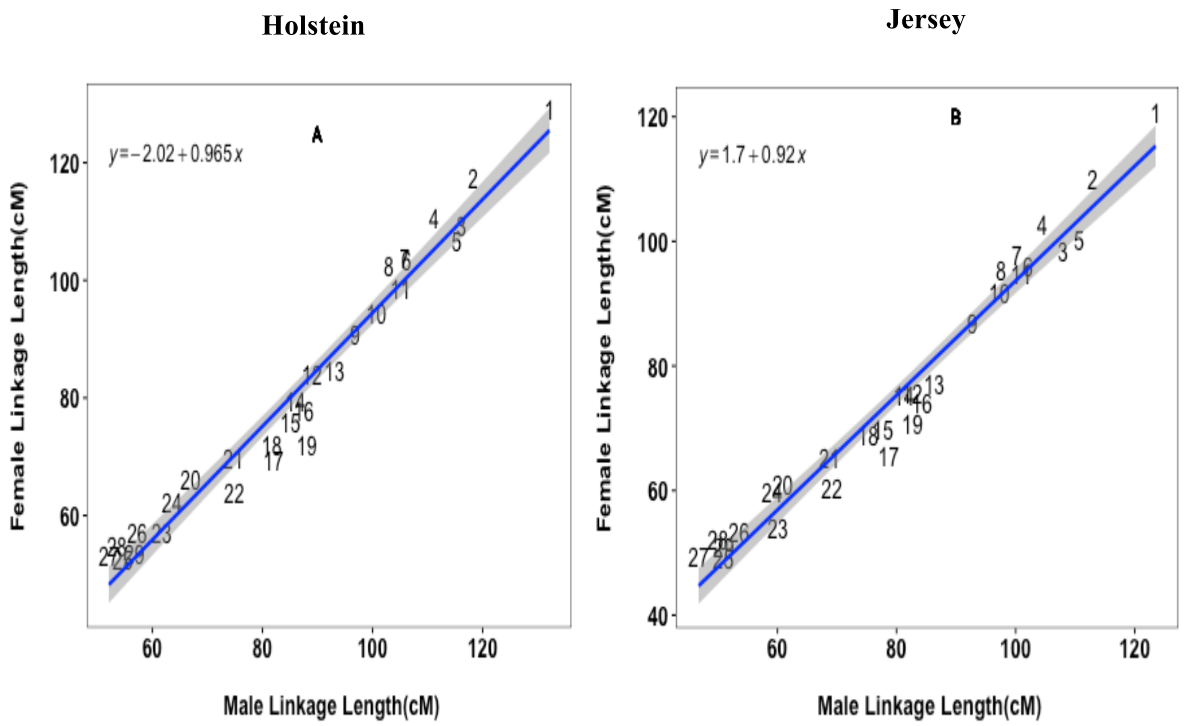

Supplemental Figure 1. The ratio of female to male in linkage map lengths (cM). Points are chromosome numbers. Lines and the grey shaded areas indicate the regression slopes and standard errors, respectively, excluding the X chromosome. A .in Holsteins; B. in Jerseys.

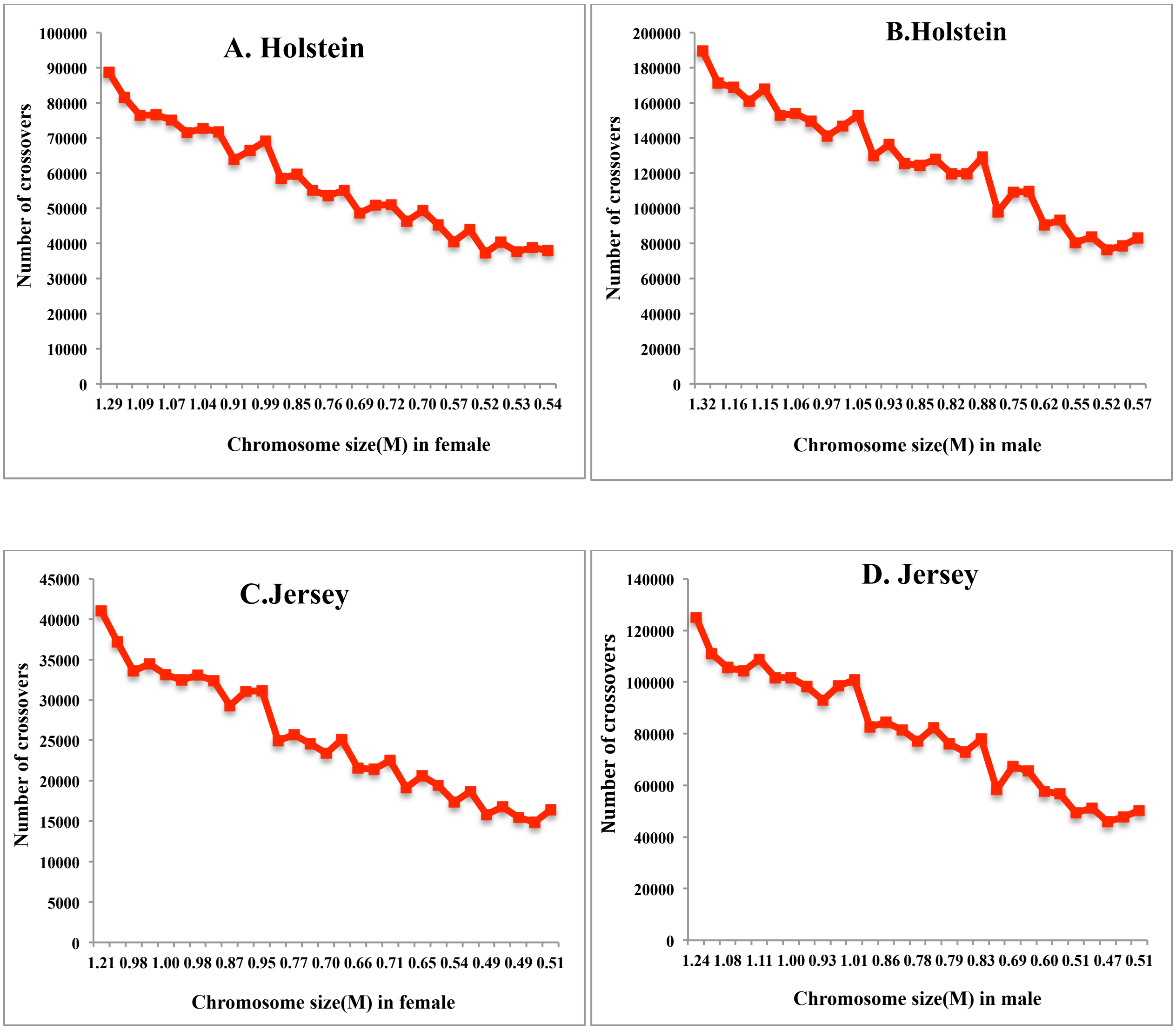

Supplemental Figure 2. The trends of number of crossovers with chromosome size in female and male. A in female and B in male were from Holstein. C in female and D in male were from Jersey.

Holstein

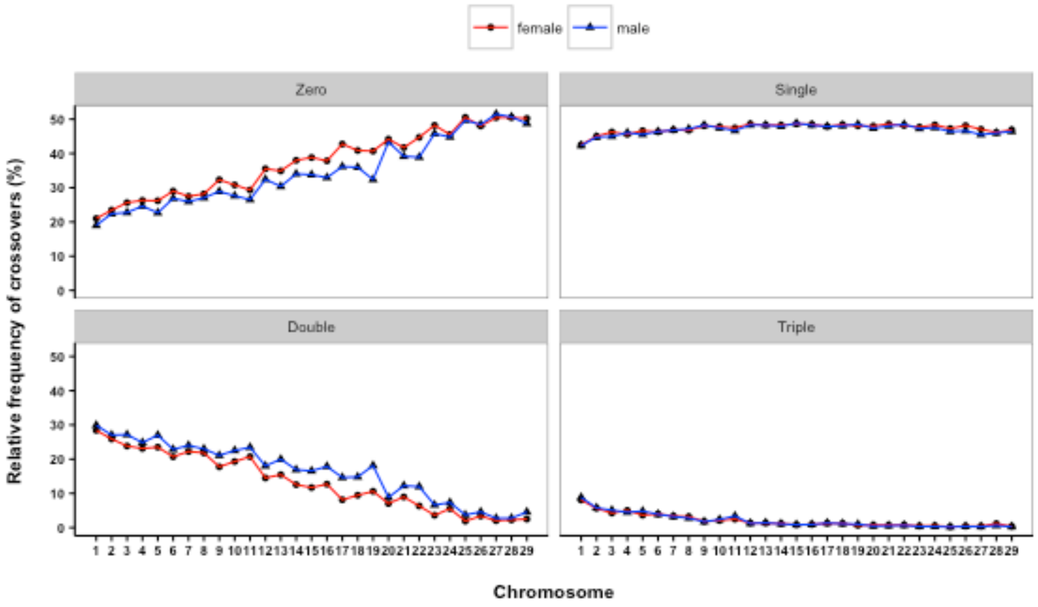

Jersey

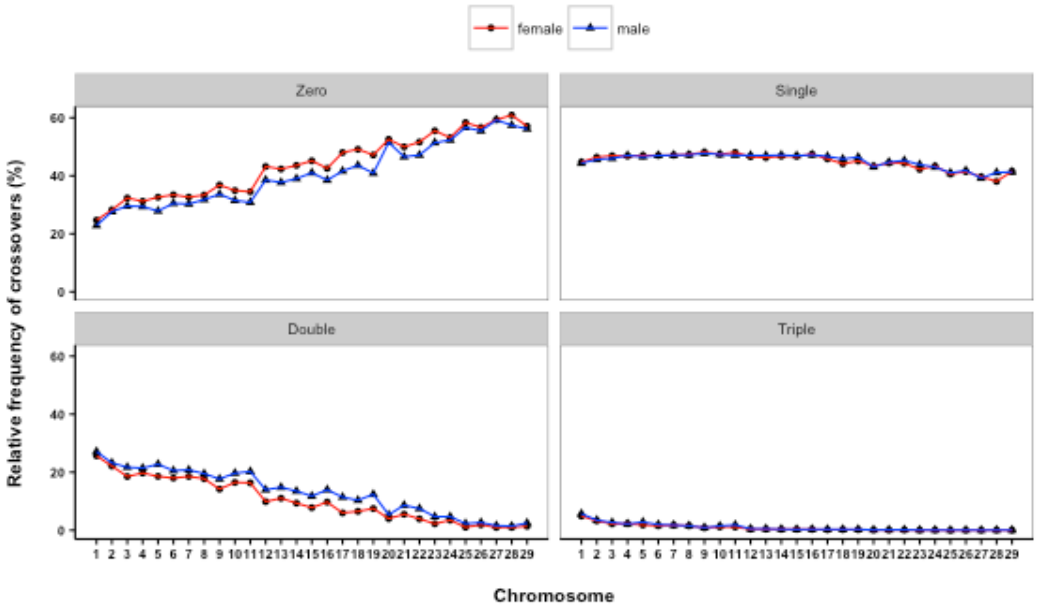

Supplemental Figure 3. Relative frequency of Zero, Single, Double, Triple crossovers on each chromosomes. Top = Holstein; Bottom = Jersey.

A: Holstein **B: Jersey**

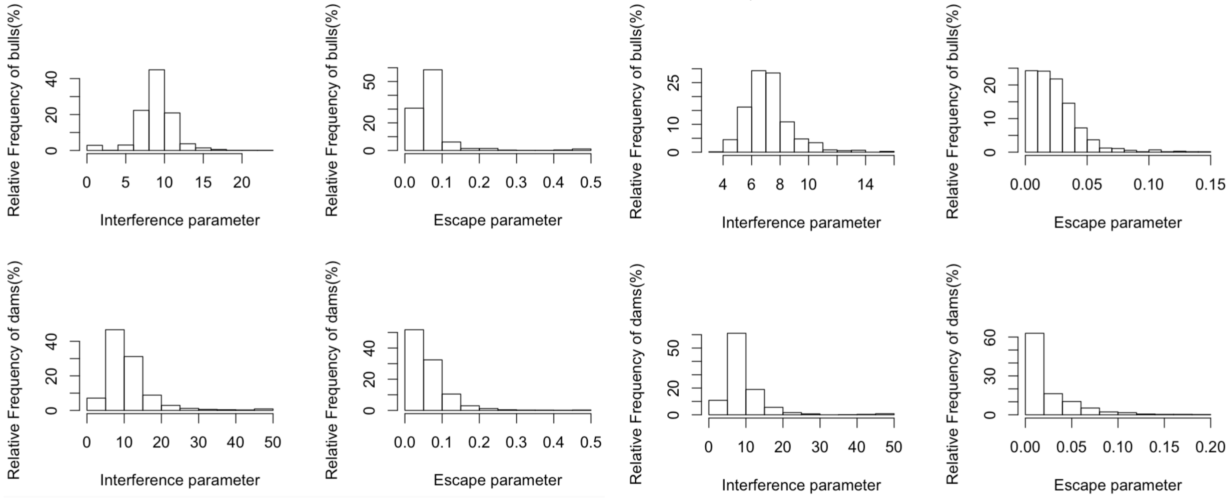

Supplemental Figure 4. The relative frequency of bulls and cows in both of interference and escape parameters. A) Relative frequency of interference and escape parameters in bulls and cows for Holstein. B) Relative frequency of interference and escape parameters in bulls and cows for Jersey. Left columns are the relative frequency of interference parameter in bulls and cows. Right column are the relative frequency of escape parameter in bulls and cows

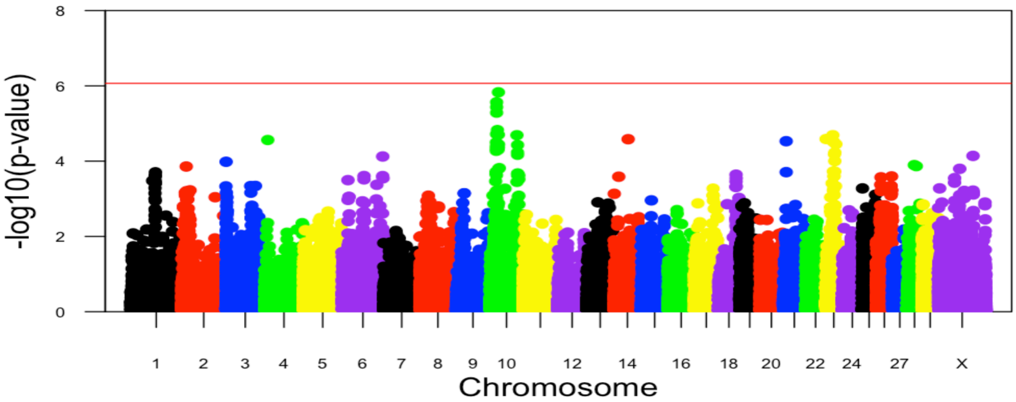

Supplemental Figure 5. Manhattan plot of the GWAS of the interference parameter for males in Jersey. Different colors were used to distinguish the 30 chromosomes. The genome-wide significance level of 8.3×10^{-7} was shown by the red horizontal solid line.

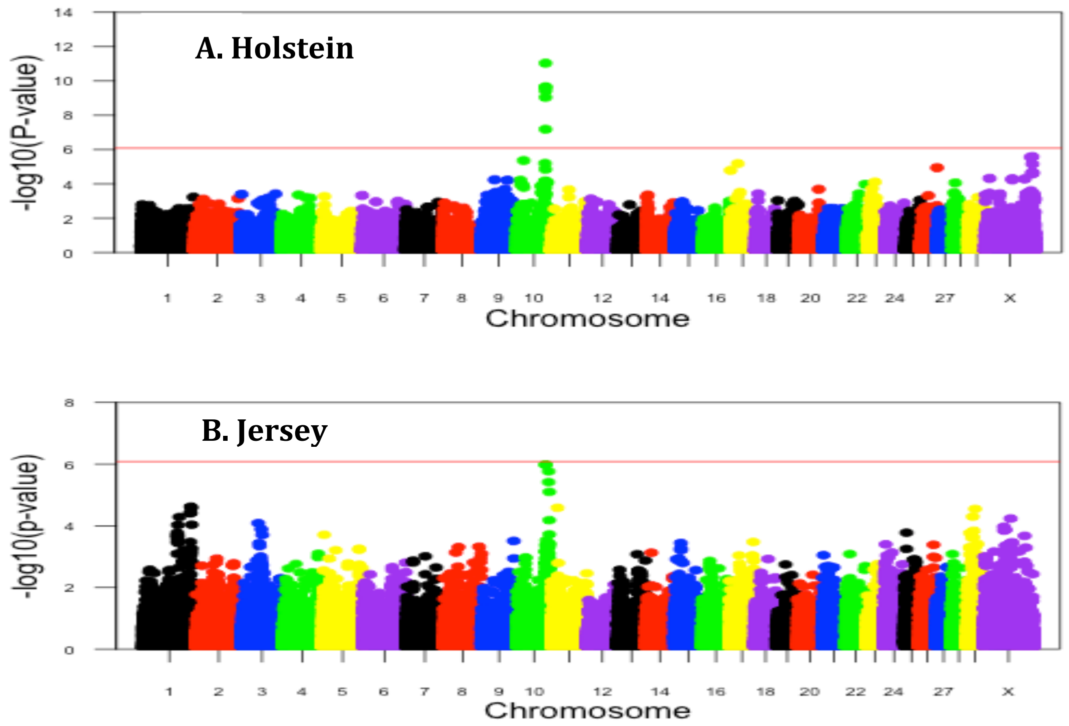

Supplemental Figure 6. Manhattan plot of the meta-GWAS of interference parameter for males (A) and for females (B) in both of Holstein and Jersey. Different colors were used to distinguish the 30 chromosomes. The genome-wide significance level of 8.3×10^{-7} was shown by the red horizontal solid line.

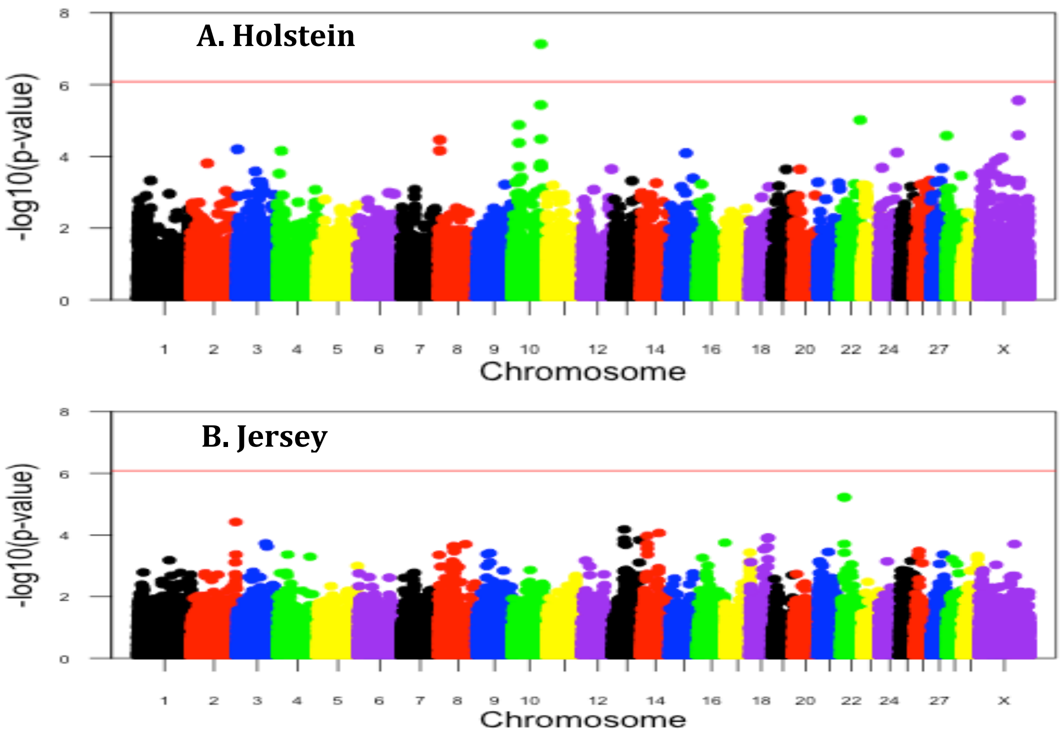

Supplemental Figure 7. Manhattan plots of the GWAS of inter-crossover distance in males (A) and females (B) in both of Holstein and Jersey. Different colors were used to distinguish the 30 chromosomes. The genome-wide significance level of 8.3×10^{-7} was shown by the red horizontal solid line.

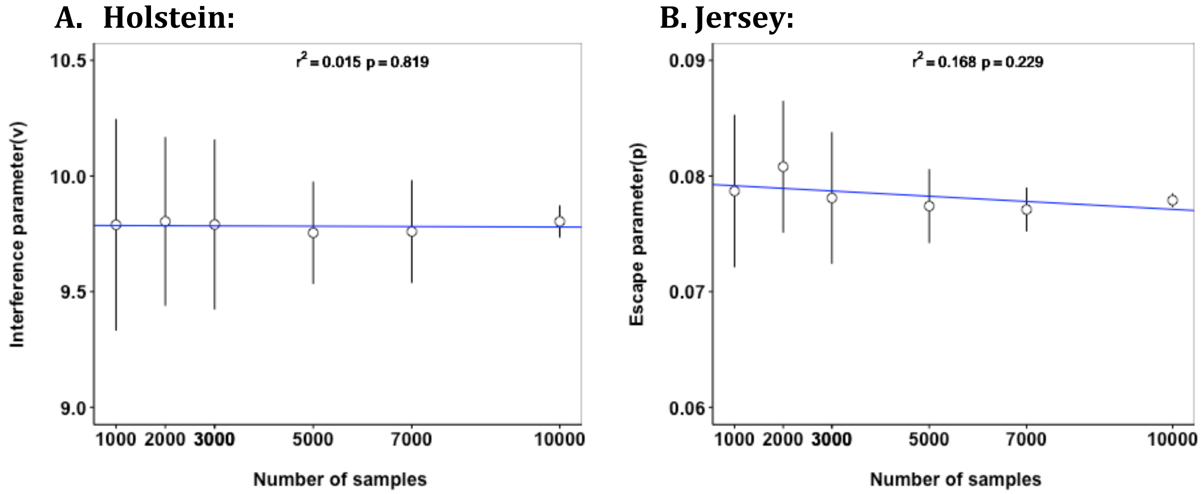

Supplemental Figure 8. Estimates of interference (A) and escape parameters (B) from different samples size in Holstein. Error bars show 95% confidence intervals.

Supplemental Tables

Supplemental Table 1. Distribution of crossover counts across 29 autosomes in 71,687 female meioses for Holstein. The values in bracket are the relative proportions of zero, single, double and triple crossovers in progeny of dams.

Chromosome	Crossover counts				Map length(M)
	0	1	2	3	
1	15053 (21.00%)	30481 (42.52%)	20367 (28.41%)	5786 (8.07%)	1.2912
2	16819 (23.46%)	32259 (45.00%)	18563 (25.89%)	4046 (5.64%)	1.1733
3	18370 (25.63%)	33175 (46.28%)	17091 (23.84%)	3051 (4.26%)	1.0930
4	18848 (26.29%)	32678 (45.58%)	16576 (23.12%)	3585 (5.00%)	1.1062
5	18777 (26.19%)	33440 (46.65%)	16848 (23.5%)	2622 (3.66%)	1.0674
6	20794 (29.01%)	33204 (46.32%)	14846 (20.71%)	2843 (3.97%)	1.0343
7	19696 (27.47%)	33624 (46.90%)	15948 (22.25%)	2419 (3.37%)	1.0376
8	20203 (28.18%)	33543 (46.79%)	15644 (21.82%)	2297 (3.20%)	1.0267
9	23175 (32.33%)	34472 (48.09%)	12740 (17.77%)	1300 (1.81%)	0.9095
10	22073 (30.79%)	34274 (47.81%)	13840 (19.31%)	1500 (2.09%)	0.9426
11	21029 (29.33%)	34023 (47.46%)	14856 (20.72%)	1779 (2.48%)	0.9873
12	25472 (35.53%)	34850 (48.61%)	10406 (14.52%)	959 (1.34%)	0.8392
13	25028 (34.91%)	34620 (48.29%)	11084 (15.46%)	955 (1.33%)	0.8465
14	27235 (37.99%)	34605 (48.27%)	9002 (12.56%)	845 (1.18%)	0.7939
15	27831 (38.82%)	34862 (48.63%)	8384 (11.70%)	610 (0.85%)	0.7581
16	27139 (37.86%)	34773 (48.51%)	9100 (12.69%)	675 (0.94%)	0.7800
17	30648 (42.75%)	34309 (47.86%)	5839 (8.15%)	891 (1.24%)	0.6940
18	29307 (40.88%)	34731 (48.45%)	6774 (9.45%)	875 (1.22%)	0.7196
19	29163 (40.68%)	34456 (48.06%)	7601 (10.60%)	467 (0.65%)	0.7200
20	31673 (44.18%)	34408 (48.00%)	5055 (7.05%)	551 (0.77%)	0.6626
21	29902 (41.71%)	34838 (48.60%)	6419 (8.95%)	528 (0.74%)	0.6981
22	32052 (44.71%)	34529 (48.17%)	4543 (6.34%)	563 (0.79%)	0.6393
23	34559 (48.21%)	34184 (47.69%)	2600 (3.63%)	344 (0.48%)	0.5703
24	32654 (45.55%)	34660 (48.35%)	3974 (5.54%)	399 (0.56%)	0.6222
25	36239 (50.55%)	33886 (47.27%)	1364 (1.90%)	198 (0.28%)	0.5244
26	34439 (48.04%)	34508 (48.14%)	2424 (3.38%)	316 (0.44%)	0.5705
27	36177 (50.47%)	33740 (47.07%)	1469 (2.05%)	301 (0.42%)	0.5313
28	36178 (50.47%)	33061 (46.12%)	1620 (2.26%)	828 (1.16%)	0.5477
29	36016 (50.24%)	33627 (46.91%)	1791 (2.50%)	253 (0.35%)	0.5362

Supplemental Table 2. Distribution of crossover counts across 29 autosomes in 147,327 male meioses for Holstein. The values in bracket are the relative proportions of zero, single, double and triple crossovers in progeny of sires.

Chromosome	Crossover counts				Map length(M)
	0	1	2	3	
1	27979(18.99%)	62309(42.29%)	44049(29.90%)	12990(8.82%)	1.3214
2	33009(22.41%)	65956(44.77%)	39862(27.06%)	8500(5.77%)	1.1830
3	33501(22.74%)	66303(45.00%)	40016(27.16%)	7507(5.10%)	1.1619
4	36265(24.62%)	67840(46.05%)	36603(24.84%)	6619(4.49%)	1.1125
5	33396(22.67%)	67086(45.54%)	39752(26.98%)	7093(4.81%)	1.1529
6	39590(26.87%)	68329(46.38%)	33666(22.85%)	5742(3.90%)	1.0618
7	38236(25.95%)	68955(46.80%)	35378(24.01%)	4758(3.23%)	1.0584
8	39820(27.03%)	69531(47.20%)	33877(22.99%)	4099(2.78%)	1.0296
9	42594(28.91%)	71098(48.26%)	31070(21.09%)	2565(1.74%)	0.9685
10	40698(27.62%)	69936(47.47%)	33196(22.53%)	3497(2.37%)	1.0067
11	39066(26.52%)	68760(46.67%)	34519(23.43%)	4982(3.38%)	1.0509
12	47783(32.43%)	71202(48.33%)	26535(18.01%)	1807(1.23%)	0.8903
13	44744(30.37%)	71110(48.27%)	29423(19.97%)	2050(1.39%)	0.9306
14	50139(34.03%)	70644(47.95%)	24928(16.92%)	1616(1.10%)	0.8602
15	49794(33.8%)	71883(48.79%)	24420(16.58%)	1230(0.83%)	0.8510
16	48534(32.94%)	71133(48.28%)	26329(17.87%)	1331(0.90%)	0.8750
17	53283(36.17%)	70533(47.88%)	21559(14.63%)	1952(1.32%)	0.8203
18	52992(35.97%)	70836(48.08%)	21820(14.81%)	1679(1.14%)	0.817
19	47703(32.38%)	71472(48.51%)	26694(18.12%)	1458(0.99%)	0.8809
20	63865(43.35%)	69784(47.37%)	13011(8.83%)	667(0.45%)	0.6696
21	57721(39.18%)	70773(48.04%)	18027(12.24%)	806(0.55%)	0.7471
22	57296(38.89%)	71419(48.48%)	17635(11.97%)	977(0.66%)	0.7487
23	67472(45.8%)	69657(47.28%)	9729(6.60%)	469(0.32%)	0.6172
24	66010(44.81%)	70023(47.53%)	10808(7.34%)	486(0.33%)	0.6358
25	73193(49.68%)	68382(46.42%)	5472(3.71%)	280(0.19%)	0.5463
26	71356(48.43%)	68692(46.63%)	6762(4.59%)	517(0.35%)	0.5726
27	75855(51.49%)	67039(45.5%)	4030(2.74%)	403(0.27%)	0.5210
28	74666(50.68%)	67712(45.96%)	4070(2.76%)	879(0.60%)	0.5358
29	71788(48.73%)	68421(46.44%)	6728(4.57%)	390(0.26%)	0.5673

Supplemental Table 3. Distribution of crossover counts across 29 autosomes in 37,008 female meioses for Jersey. The values in bracket were the relative proportions of zero, single, double and triple crossovers in progeny of dams.

Chromosome	Crossover counts				Map length(M)
	0	1	2	3	
1	9139 (24.69%)	16551 (44.72%)	9502 (25.68%)	1816 (4.91%)	1.2084
2	10446 (28.23%)	17166 (46.38%)	8194 (22.14%)	1202 (3.25%)	1.1019
3	11967 (32.34%)	17358 (46.90%)	6861 (18.54%)	822 (2.22%)	0.9842
4	11547 (31.20%)	17311 (46.78%)	7307 (19.74%)	843 (2.28%)	1.0287
5	12063 (32.6%)	17395 (47.00%)	6880 (18.59%)	670 (1.81%)	1.0020
6	12367 (33.42%)	17392 (47.00%)	6665 (18.01%)	584 (1.58%)	0.9610
7	12071 (32.62%)	17431 (47.10%)	6867 (18.56%)	639 (1.73%)	0.9773
8	12338 (33.34%)	17514 (47.32%)	6581 (17.78%)	575 (1.55%)	0.9537
9	13623 (36.81%)	17814 (48.14%)	5252 (14.19%)	319 (0.86%)	0.8685
10	12924 (34.92%)	17549 (47.42%)	6109 (16.51%)	426 (1.15%)	0.9166
11	12768 (34.5%)	17776 (48.03%)	6047 (16.34%)	417 (1.13%)	0.9496
12	15981 (43.18%)	17233 (46.57%)	3652 (9.87%)	142 (0.38%)	0.7568
13	15661 (42.32%)	17118 (46.25%)	4085 (11.04%)	144 (0.39%)	0.7710
14	16116 (43.55%)	17300 (46.75%)	3467 (9.37%)	125 (0.34%)	0.7536
15	16716 (45.17%)	17283 (46.7%)	2883 (7.79%)	126 (0.34%)	0.6987
16	15748 (42.55%)	17547 (47.41%)	3596 (9.72%)	117 (0.32%)	0.7419
17	17763 (48%)	16960 (45.83%)	2217 (5.99%)	68 (0.18%)	0.6573
18	18182 (49.13%)	16325 (44.11%)	2416 (6.53%)	85 (0.23%)	0.6886
19	17455 (47.17%)	16695 (45.11%)	2777 (7.5%)	81 (0.22%)	0.7085
20	19448 (52.55%)	16030 (43.31%)	1485 (4.01%)	45 (0.12%)	0.6106
21	18489 (49.96%)	16436 (44.41%)	2049 (5.54%)	34 (0.09%)	0.6543
22	19107 (51.63%)	16407 (44.33%)	1453 (3.93%)	41 (0.11%)	0.6053
23	20544 (55.51%)	15613 (42.19%)	836 (2.26%)	15 (0.04%)	0.5408
24	19658 (53.12%)	16032 (43.32%)	1294 (3.5%)	24 (0.06%)	0.5978
25	21586 (58.33%)	15026 (40.6%)	393 (1.06%)	3 (0.01%)	0.4925
26	20964 (56.65%)	15329 (41.42%)	700 (1.89%)	15 (0.04%)	0.5331
27	21951 (59.31%)	14675 (39.65%)	366 (0.99%)	16 (0.04%)	0.4932
28	22532 (60.88%)	14084 (38.06%)	376 (1.02%)	16 (0.04%)	0.5221
29	21119 (57.07%)	15375 (41.55%)	506 (1.37%)	8 (0.02%)	0.5089

Supplemental Table 4. Distribution of crossover counts across 29 chromosomes in 108,163 male meioses for Jersey. The values in bracket were the relative proportions of zero, single, double and triple crossovers in progeny of sires.

Chromosome	Crossover counts				Map length(M)
	0	1	2	3	
1	24718(22.85%)	47952(44.33%)	29340(27.13%)	6153(5.69%)	1.2350
2	29892(27.64%)	49364(45.64%)	25126(23.23%)	3781(3.50%)	1.1291
3	31990(29.58%)	49740(45.99%)	23505(21.73%)	2928(2.71%)	1.0797
4	31780(29.38%)	50823(46.99%)	23181(21.43%)	2379(2.20%)	1.0450
5	30112(27.84%)	50346(46.55%)	24626(22.77%)	3079(2.85%)	1.1065
6	32980(30.49%)	50821(46.99%)	22234(20.56%)	2128(1.97%)	1.0208
7	32753(30.28%)	50948(47.10%)	22512(20.81%)	1950(1.80%)	1.0021
8	34365(31.77%)	51022(47.17%)	21102(19.51%)	1674(1.55%)	0.9760
9	36392(33.65%)	51633(47.74%)	19155(17.71%)	983(0.91%)	0.9274
10	34061(31.49%)	51207(47.34%)	21291(19.68%)	1604(1.48%)	0.9729
11	33355(30.84%)	50915(47.07%)	21898(20.25%)	1995(1.84%)	1.0094
12	41676(38.53%)	50865(47.03%)	15095(13.96%)	527(0.49%)	0.8264
13	40779(37.7%)	50814(46.98%)	16024(14.81%)	546(0.50%)	0.8633
14	42178(38.99%)	50968(47.12%)	14605(13.50%)	412(0.38%)	0.8127
15	44377(41.03%)	50737(46.91%)	12746(11.78%)	303(0.28%)	0.7772
16	41632(38.49%)	51047(47.19%)	15118(13.98%)	366(0.34%)	0.8428
17	45014(41.62%)	50471(46.66%)	12344(11.41%)	334(0.31%)	0.7866
18	47119(43.56%)	49517(45.78%)	11199(10.35%)	328(0.30%)	0.7520
19	44208(40.87%)	50208(46.42%)	13410(12.40%)	337(0.31%)	0.8269
20	55692(51.49%)	46563(43.05%)	5797(5.36%)	111(0.10%)	0.6089
21	50334(46.54%)	48339(44.69%)	9348(8.64%)	142(0.13%)	0.6877
22	50964(47.12%)	48942(45.25%)	8098(7.49%)	159(0.15%)	0.6916
23	55612(51.41%)	47409(43.83%)	5070(4.69%)	72(0.07%)	0.6006
24	56556(52.29%)	46486(42.98%)	5066(4.68%)	55(0.05%)	0.5915
25	61289(56.66%)	44350(41.00%)	2490(2.30%)	34(0.03%)	0.5091
26	59994(55.47%)	45219(41.81%)	2891(2.67%)	59(0.05%)	0.5356
27	64030(59.20%)	42393(39.19%)	1727(1.60%)	13(0.01%)	0.4677
28	62108(57.42%)	44481(41.12%)	1526(1.41%)	48(0.04%)	0.5004
29	60732(56.15%)	44675(41.30%)	2717(2.51%)	39(0.04%)	0.5099

Supplemental Table 5. The relative frequency (%) of two or more crossovers in Holstein and Jersey.

Chromosome	Holstein		Jersey		HO:JE	
	Female	Male	Female	Male	Ratio of female	Ratio of male
1	36.48	38.72	30.58	32.81	1.19	1.18
2	31.54	32.83	25.39	26.73	1.24	1.23
3	28.10	32.26	20.76	24.44	1.35	1.32
4	28.12	29.34	22.02	23.63	1.28	1.24
5	27.16	31.80	20.40	25.61	1.33	1.24
6	24.68	26.75	19.59	22.52	1.26	1.19
7	25.62	27.24	20.28	22.62	1.26	1.20
8	25.03	25.78	19.34	21.06	1.29	1.22
9	19.59	22.83	15.05	18.62	1.30	1.23
10	21.40	24.91	17.66	21.17	1.21	1.18
11	23.21	26.81	17.47	22.09	1.33	1.21
12	15.85	19.24	10.25	14.44	1.55	1.33
13	16.79	21.36	11.43	15.32	1.47	1.39
14	13.74	18.02	9.71	13.88	1.42	1.30
15	12.55	17.41	8.13	12.06	1.54	1.44
16	13.64	18.77	10.03	14.32	1.36	1.31
17	9.39	15.96	6.17	11.72	1.52	1.36
18	10.67	15.95	6.76	10.66	1.58	1.50
19	11.25	19.11	7.72	12.71	1.46	1.50
20	7.82	9.28	4.13	5.46	1.89	1.70
21	9.69	12.78	5.63	8.77	1.72	1.46
22	7.12	12.63	4.04	7.63	1.76	1.65
23	4.11	6.92	2.30	4.75	1.79	1.46
24	6.10	7.67	3.56	4.73	1.71	1.62
25	2.18	3.90	1.07	2.33	2.04	1.67
26	3.82	4.94	1.93	2.73	1.98	1.81
27	2.47	3.01	1.03	1.61	2.39	1.87
28	3.41	3.36	1.06	1.46	3.22	2.31
29	2.85	4.83	1.39	2.55	2.05	1.90
mean	15.32	18.43	11.20	14.08	1.60	1.45

Supplemental Table 6. Estimates of interference and escape parameters on each chromosome for female and male in Holstein.

Chromosome	Interference parameter				Escape parameter		L_{female}	L_{male}
	v_{female}	sd_{femal}	v_{male}	sd_{male}	p_{female}	p_{male}		
1	8.4702	0.0135	8.2771	0.0062	0.0944	0.0999	1.2912	1.3214
2	8.2381	0.0136	7.9271	0.0060	0.0853	0.0761	1.1733	1.183
3	8.1513	0.0141	7.2162	0.0046	0.0661	0.0512	1.093	1.1619
4	9.5248	0.0248	8.8468	0.0099	0.1583	0.1341	1.1062	1.1125
5	8.5267	0.016	7.7356	0.0061	0.0714	0.0641	1.0674	1.1529
6	9.0066	0.0233	8.6921	0.0096	0.1463	0.1357	1.0343	1.0618
7	8.1867	0.0165	7.4905	0.0062	0.0914	0.0722	1.0376	1.0584
8	8.5741	0.017	8.1558	0.0075	0.0824	0.0701	1.0267	1.0296
9	8.7029	0.0203	8.8957	0.0106	0.075	0.0662	0.9095	0.9685
10	8.0909	0.0165	7.5326	0.0061	0.0643	0.0476	0.9426	1.0067
11	8.8935	0.0196	8.2175	0.0079	0.0863	0.0941	0.9873	1.0509
12	9.8103	0.0338	9.37	0.0128	0.0892	0.075	0.8392	0.8903
13	9.4918	0.0284	8.251	0.0082	0.0716	0.0531	0.8465	0.9306
14	10.4953	0.0482	9.1639	0.0139	0.0992	0.0681	0.7939	0.8602
15	9.8287	0.0382	8.4859	0.0102	0.073	0.0531	0.7581	0.851
16	9.7676	0.0352	8.0966	0.0082	0.0751	0.0488	0.78	0.875
17	12.6922	0.1082	10.0184	0.0211	0.1304	0.1061	0.694	0.8203
18	13.94	0.1292	8.9504	0.0127	0.138	0.076	0.7196	0.817
19	10.0178	0.0419	7.4066	0.0061	0.0654	0.0399	0.72	0.8809
20	12.2664	0.1042	9.5088	0.033	0.0989	0.0818	0.6626	0.6696
21	10.5899	0.0557	9.7147	0.0187	0.0824	0.0604	0.6981	0.7471
22	15.0447	0.1911	11.312	0.0277	0.1121	0.0837	0.6393	0.7487
23	16.7165	0.3469	11.3275	0.043	0.0919	0.0715	0.5703	0.6172
24	14.067	0.1666	9.8328	0.0352	0.0877	0.0768	0.6222	0.6358
25	47.8346	11.0619	18.7867	0.2804	0.0866	0.0837	0.5244	0.5463
26	19.1931	0.5221	13.6051	0.1122	0.0968	0.1094	0.5705	0.5726
27	44.9032	10.1088	50.0000	12.3312	0.0994	0.1229	0.5313	0.521
28	40.3072	8.8968	43.0031	7.6867	0.1715	0.1503	0.5477	0.5358
29	26.6136	2.0512	12.5159	0.1032	0.0915	0.0873	0.5362	0.5673

Supplemental Table 7. Estimates of interference and escape interference on each chromosome for female and male in Jersey.

Chromosome	Interference parameter				Escape parameter		L_{female}	L_{male}
	v_{female}	sd_{female}	v_{male}	sd_{male}	p_{female}	p_{male}		
1	6.6860	0.0149	6.312	0.0039	0.0195	0.0198	1.2084	1.235
2	6.4713	0.0156	5.9535	0.0042	0.0207	0.0166	1.1019	1.1291
3	6.1629	0.0178	6.2892	0.0048	0.0225	0.0143	0.9842	1.0797
4	7.2353	0.0219	6.4055	0.005	0.0365	0.0267	1.0287	1.045
5	6.6147	0.0196	6.2136	0.0046	0.0242	0.0173	1.002	1.1065
6	7.2462	0.0255	6.4179	0.0058	0.0426	0.035	0.961	1.0208
7	6.2652	0.019	6.054	0.0043	0.0256	0.0134	0.9773	1.0021
8	6.7625	0.0195	6.0383	0.0047	0.0239	0.0176	0.9537	0.976
9	6.9464	0.0252	6.402	0.006	0.0217	0.0165	0.8685	0.9274
10	7.0975	0.0271	6.4418	0.0055	0.0255	0.0148	0.9166	0.9729
11	7.2851	0.028	6.7386	0.0067	0.0309	0.0367	0.9496	1.0094
12	7.2497	0.0405	6.5858	0.0076	0.0256	0.0178	0.7568	0.8264
13	7.0006	0.0328	6.7095	0.0079	0.021	0.0165	0.771	0.8633
14	8.0843	0.0521	6.988	0.0087	0.0312	0.0177	0.7536	0.8127
15	8.1108	0.0594	7.1766	0.0105	0.0325	0.018	0.6987	0.7772
16	7.9364	0.0467	6.6308	0.0083	0.0223	0.0183	0.7419	0.8428
17	8.9149	0.0973	7.5635	0.0123	0.0345	0.0225	0.6573	0.7866
18	9.1354	0.1019	6.986	0.0116	0.0408	0.0191	0.6886	0.752
19	8.2021	0.0712	6.7221	0.0097	0.0319	0.016	0.7085	0.8269
20	10.575	0.2002	6.9921	0.0182	0.0446	0.0289	0.6106	0.6089
21	8.4442	0.0857	6.494	0.0103	0.0298	0.0156	0.6543	0.6877
22	10.4304	0.1677	8.0019	0.0214	0.0362	0.0269	0.6053	0.6916
23	11.1212	0.4084	8.0864	0.0272	0.035	0.0276	0.5408	0.6006
24	11.4805	0.3351	7.3131	0.0202	0.0401	0.0247	0.5978	0.5915
25	21.883	3.767	10.539	0.103	0.0407	0.0397	0.4925	0.5091
26	11.8205	0.3454	9.2452	0.0646	0.0336	0.0393	0.5331	0.5356
27	19.6387	2.1022	9.8799	0.0993	0.0386	0.0308	0.4932	0.4677
28	17.5138	1.7216	19.0518	0.8984	0.0532	0.0525	0.5221	0.5004
29	15.8311	0.9987	8.4196	0.0455	0.0332	0.0291	0.5089	0.5099

Supplemental Table 8. SNPs associated with interference parameter in male for Holstein and Jersey.

SNP_rs	SNP	Chr	Position	Freq	Beta	*P*-value
rs43640523	BTA-78285-no-rs	10	86717378	0.53	0.54	9.2×10^{-12}
rs43643710	BTB-00438757	10	86956123	0.36	0.53	2.2×10^{-10}
rs41257023	Hapmap57084-ss46526565	10	87016969	0.64	-0.53	2.3×10^{-10}
rs43640475	BTB-00438922	10	86679535	0.36	0.52	3.8×10^{-10}
rs41591947	UA-IFASA-7857	10	86774733	0.44	-0.49	9.0×10^{-10}
rs43644595	ARS-BFGL-NGS-113063	10	86615332	0.63	0.45	6.6×10^{-8}

Supplemental Table 9. Estimates of interference and escape on each maternal age group for Holstein.

Maternal Age (month)	n_{all}	v_{all}	sd_{all}	p_{all}	n_{679}	v_{679}	sd_{679}	p_{679}
25~35	12469	9.7728	0.0063	0.0776	679	8.4723	1.2845	0.0705
35~45	13919	9.9626	0.0066	0.0812	679	9.5446	1.9686	0.0777
45~55	11204	9.8546	0.0075	0.0801	679	9.5075	1.7337	0.0766
55~65	6180	10.0042	0.0149	0.0855	679	9.6628	1.8261	0.0819
65~75	3222	9.7805	0.0262	0.0654	679	8.1066	1.535	0.0692
75~85	1678	9.6705	0.0486	0.0603	679	7.9939	0.8983	0.035
85~95	722	9.158	0.0917	0.0586	679	7.4633	0.9401	0.0376
95~180	696	10.006	0.1394	0.0655	679	7.9399	0.7098	0.0237

Supplemental Table 10. Distribution of crossover counts in each maternal age groups for Holstein. Min(N_i(x)) is the statistic from chromosome 1.

Maternal Age (month)	Number of Individuals	Crossover Counts			
		0	1	2	3
25~35	12469	2544(20.40%)	5319(42.66%)	3642(29.21%)	964(7.73%)
35~45	13919	2848(20.46%)	6103(43.85%)	3945(28.34%)	1023(7.35%)
45~55	11204	2466(22.01%)	4756(42.45%)	3131(27.95%)	851(7.6%)
55~65	6180	1349(21.83%)	2643(42.77%)	1720(27.83%)	468(7.57%)
65~75	3222	750(23.28%)	1342(41.65%)	897(27.84%)	233(7.23%)
75~85	1678	375(22.35%)	709(42.25%)	478(28.49%)	116(6.91%)
85~95	722	154(21.33%)	312(43.21%)	211(29.22%)	45(6.23%)
95~180	696	143(20.55%)	280(40.23%)	226(32.47%)	47(6.75%)
Min(N_i(x))	679	143(21.06%)	280(41.24%)	211(31.08%)	45(6.63%)
